# Long-term survival benefit of male and multimorbid COVID-19 patients with 5-day remdesivir treatment

**DOI:** 10.7189/jogh.12.05031

**Published:** 2022-08-31

**Authors:** Lorinc Polivka, Jozsef Gajdacsi, Levente Fazekas, Szilvia Sebok, Eniko Barczi, Edit Hidvegi, Zoltan Sutto, Elek Dinya, Pal Maurovich-Horvat, Attila J Szabo, Bela Merkely, Veronika Müller

**Affiliations:** 1 Department of Pulmonology, Semmelweis University, Budapest, Hungary; 2 Clinical Center, Semmelweis University, Budapest, Hungary; 3 Heart and Vascular Center, Semmelweis University, Budapest, Hungary; 4 University Pharmacy Department of Pharmacy Administration, Semmelweis University, Budapest, Hungary; 5 Institute of Digital Health Sciences, Semmelweis University, Budapest, Hungary; 6 Medical Imaging Centre, Semmelweis University, Budapest, Hungary; 7 1st Department of Pediatrics, Semmelweis University, Budapest, Hungary

## Abstract

**Background:**

Treatment of the coronavirus disease (COVID-19) is still challenging due to the lack of evidence-based treatment protocols and continuously changing epidemiological situations and vaccinations. Remdesivir (RDV) is among the few antiviral medications with confirmed efficacy for specific patient groups. However, real-world data on long-term outcomes for a short treatment course are scarce.

**Methods:**

This retrospective observational cohort study included real-life data collected during the second and third wave of the COVID-19 pandemic in Hungary (September 1, 2020-April 30, 2021) from inpatients at a University Center (n = 947). Participants consisted of two propensity score-matched cohorts (370/370 cases): Group RDV including patients receiving RDV and supplementary oxygen and Group standard of care (SOC) as control. The primary outcome was the effect of 5-day RDV treatment on 30- and 60-day all-cause mortality. Multivariate analyses were performed to assess the effect of RDV by different covariates.

**Results:**

Group RDV included significantly more patients from the alpha variant wave, with greater frequency of comorbidities diabetes and anemia, and larger degree of parenchymal involvement. All-cause mortality at 30- and 60-day were significantly lower in Group RDV compared to Group SOC. Significant risk reduction of 60-day all-cause mortality was observed for RDV treatment in men and patients with COPD or multiple comorbidities.

**Conclusions:**

Hospitalized COVID-19 patients with 5-day RDV treatment had significantly lower 30- and 60-day all-cause mortality, despite their more severe clinical condition. Men and patients with multiple comorbidities, including COPD, profited the most from RDV treatment in the long term. Due to the ongoing COVID-19 pandemic, effective treatment regimens are needed for hospitalized patients.

Numerous antiviral agents have been tested for clinical effectiveness against the SARS-CoV-2 virus. Based on the ACTT-1 trial [1], remdesivir (RDV) was first approved on June 25, 2020, by the European Medicines Agency [2] and is conditionally recommended for treatment of hospitalized patients who require supplemental oxygen but not mechanical ventilation [3]. Other studies, including the WHO-SOLIDARITY trial [4], show less or even no benefit for the drug. The European Respiratory Society living therapeutic guidelines has not formed recommendation regarding the use of RDV, however, no difference between the 10-day and the 5-day long treatment durations regarding outcome in hospitalized patients is stated [5]. This is based on studies showing similar benefit for both RDV length interventions [6,7]. Based on the recent PINETREE trial, the use of 3-day RDV for outpatients was also beneficial [8].

It is often difficult to evaluate the real-world effect of a given medication due to constantly changing factors, including virus type, emerging clinical trial data of different treatments, vaccination availability, and others. Similarly, it is challenging to evaluate the efficacy of antiviral treatment, especially on the long term. Because of these factors, more real-world data based on clinical experience are essential to provide a base for the meta-analyses. Our aim was to perform a retrospective analysis regarding the clinical efficacy of a 5-day use of RDV treatment during the second (wild variant) and third wave (alpha variant) of the pandemic, and to identify factors influencing favorable long-term outcome of antiviral treatment.

## METHODS

### Patient selection and study design

**Figure 1:**
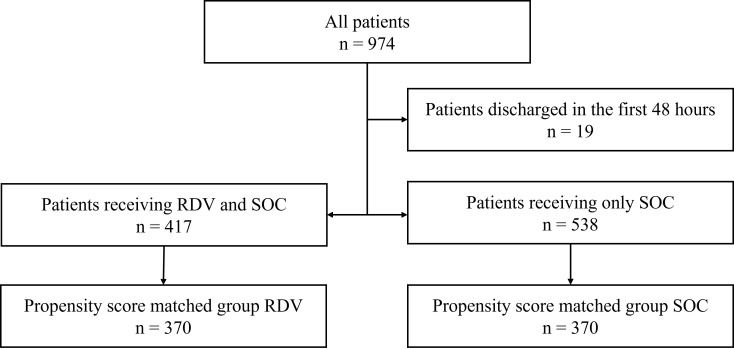
Cohort selection. RDV – remdesivir, SOC – only standard of care.

This retrospective study included PCR and/or antigen confirmed COVID-19 patients (ICD U07.10) admitted to Semmelweis University’s Department of Pulmonology between September 1, 2020, and April 30, 2021. This period covers most of the second and third wave of the pandemic in Hungary, when the predominant types of SARS-CoV2 were the wild type and alpha (B.1.1.7) variants of the virus, respectively [9]. Patients starting from January 27, 2021, were considered to relate to the third wave based on the 7-day moving average of new COVID-19 cases in Hungary. **Figure 1** describes the study population selection. Cases were divided according to treatment into patients receiving only standard of care (SOC), or RDV and SOC. Final analysis included propensity score-matched (PSM) patients assigned to Group RDV and Group SOC.

### Parameters

Age at admission, sex, medical history of comorbidities, and body mass index (BMI) was collected. During the hospital stay, the daily maximum of National Early Warning Score 2 (NEWS2]) were assigned to the cases, as well as daily maximum of oxygen dependency, to calculate the WHO’s Ordinal Scale for Clinical Improvement (WHOS) [10]. The NEWS2 is based on a simple aggregate scoring system including six simple physiological parameters which form the basis of the scoring system: respiration rate, oxygen saturation and the need of supplementary oxygen, systolic blood pressure, pulse rate, level of consciousness or new confusion, and body temperature [11]. For all patients, discharge documents were used to retrieve the Charlson comorbidity index [12], compressing age and comorbid conditions into one variable. Comorbid conditions were based on the ICD-10 classification using categories defined by Hude Quan et al. [13]. The score is a weighted index to predict risk of death within 1 year of hospitalization for patients with specific comorbid conditions. Comorbidities included malignancies (embracing all types of solid and haematological cancers), hypertension, diabetes, coronary artery disease (CAD), heart failure, bronchial asthma, chronic obstructive pulmonary disease (COPD), anaemia, and dyslipidaemia. Due to large proportion of missing body height data, BMI calculation and data on obesity were not included.

As part of the local protocol, initial low-dose chest CT was performed on the day of admission ± two days. Extent of pneumonia was classified according CO-RADS [14], including COVID-19 diagnosis confidence and percent of parenchymal involvement, according to recommendations of the American Collage or Radiology [15]. Baseline laboratory parameters were recorded, including serum levels of C-reactive protein (CRP), procalcitonin (PCT), ferritin, interleukin-6 (IL-6), prohormone of brain natriuretic peptide (ProBNP), troponin, and estimated glomerular filtration rate (eGFR), serum levels of aspartate aminotransferase (GOT), alanine aminotransferase (GPT), total protein (TP) and albumin. Additionally, red blood cell count (RBC), blood haemoglobin (Hgb), platelet count (PLT), white blood cell count (WBC) and lymphocyte count (LC) were collected. The baseline day was defined as the day of the first dose of RDV or the day of admission for patients in Group RDV and Group SOC, respectively.

### Treatment protocols

Based on the department’s SOC protocol, indication for oxygen supplementation was partial pressure of arterial oxygen (P_a_O_2_)<60 mm Hg or oxygen saturation measured by pulse oximetry (SpO_2_)≤90% at room air. Beginning oxygen flow was 2 L/min via nasal cannula and was titrated up to 15 L/min with reservoir facemask to ensure SpO_2_>90% [16]. Antiviral therapy included favipiravir or RDV. In selected cases, convalescent plasma was also administered. The standard anti-inflammatory medication was systemic glucocorticoid, while it was supplemented with baricitinib or tocilizumab in selected severe COVID-19 (WHOS 5) cases during the third wave. Subcutaneous enoxaparin sodium was applied for thromboprophylaxis, and vitamins C and D were given as supplements. Treatment protocols used for the study period are summarized in **Table 1**. It is important to note that non-invasive ventilation and high flow oxygen was avoided until February 2021 to prevent infection of unvaccinated health care personnel (HCP). Vaccination of all HCP assigned to the COVID-19 stations was completed by the end of January 2021.

**Table 1:** Treatment protocol timeline at the department

Treatment protocol I. 01 September 2020-13 October 2020	1. Favipiravir (7-10 d) per individual decision of patient and physician
	2. Oxygen supplementation, targeted SpO_2_>90% or Pao_2_≥60 mm Hg
	3. Methylprednisolone 4-16 mg
	4. Convalescent plasma therapy (in the first 72 h, in selected cases)
	5. Prophylactic LMWH (weight adjusted)
	6. Azithromycin (3 d, 500 mg/d)
	7. Histamine 2 receptor blocker or proton pump inhibitor
	8. Vitamin C 1000 mg
	9. Vitamin D 2000 IU
Treatment protocol II, 14 October 2020-14 February 2021	1. Remdesivir (5 d) according SmPC or favipiravir (7-10 d)
	2. Oxygen supplementation, targeted SpO_2_>90% or Pao_2_≥60 mm Hg
	3. Methylprednisolone 8 mg/Dexamethasone 8-16 mg
	4. Convalescent plasma therapy (in the first 72 h if needed)
	5. Prophylactic LMWH (weight adjusted)
	6. Azithromycin (3 d – 500 mg)
	7. Histamine 2 receptor blocker or proton pump inhibitor
	8. Vitamin C 1000 mg
	9. Vitamin D 2000 IUE
	10. Individual decision for tocilizumab/baricitinib/monoclonal antibodies
Treatment protocol III, 15 February 2021-28 February 2021	1. Remdesivir (5 d) according SmP
	2. Oxygen supplementation, targeted SpO_2_>90% or Pao_2_≥60 mm Hg
	3. Methylprednisolone 8 mg /Dexamethasone iv 8-16 mg
	4. Convalescent plasma therapy (in the first 72 h, in selected cases)
	5. Prophylactic LMWH (weight adjusted)
	6. Acetylsalicylic acid 1×100 mg
	7. Histamine 2 receptor blocker or proton pump inhibitor
	8. Vitamin C 1000 mg
	9. Vitamin D 2000 IU
	10. Individual decision for tocilizumab/baricitinib/monoclonal antibodies
Treatment protocol IV, 01 March 2021-31 March 2021	1. Remdesivir (5 d) according SmPC
	2. Oxygen supplementation, targeted SpO_2_>90% or Pao_2_≥60 mm Hg
	3. Dexamethasone 8 mg oral/8-16 mg iv
	4. Convalescent plasma therapy (in the first 72 h, in selected cases)
	5. Prophylactic LMWH (weight adjusted)
	6. Acetylsalicylic acid 1×100 mg
	7. Histamine 2 receptor blocker (famotidin 2×40 mg)
	8. Vitamin C 1000 mg
	9. Vitamin D 2000 IU
	10. Individual decision for tocilizumab/baricitinib/monoclonal antibodies
Treatment protocol V, 01 April 2021-30 April 2021	1. Remdesivir (5 d) according SmPC10. Specific decision for tocilizumab/baricitinib/monoclonal antibodies
	2. Oxygen supplementation, targeted SpO_2_>90% or Pao_2_≥60 mm Hg
	3. Dexamethasone 8-16 mg /iv 8-16 mg
	4. Convalescent plasma therapy (in the first 72 h, in selected cases)
	5. Prophylactic LMWH (weight adjusted)
	6. Acetylsalicylic acid 1×100 mg
	7. Histamine 2 receptor blocker (famotidin 240 mg)
	8. Vitamin C 1000 mg
	9. Vitamin D 2000 IU
	10. Individual decision for tocilizumab/baricitinib/monoclonal antibodies

SmPC – summary of product characteristics, LMWH – low molecular weight heparin, d – days, iv – intravenous, h – hours, d – days, IU – international units

### Outcomes

Primary outcome of the analysis was all-cause mortality at 30- and 60-day post-admission in Group RDV and Group SOC.

**Figure 2:**
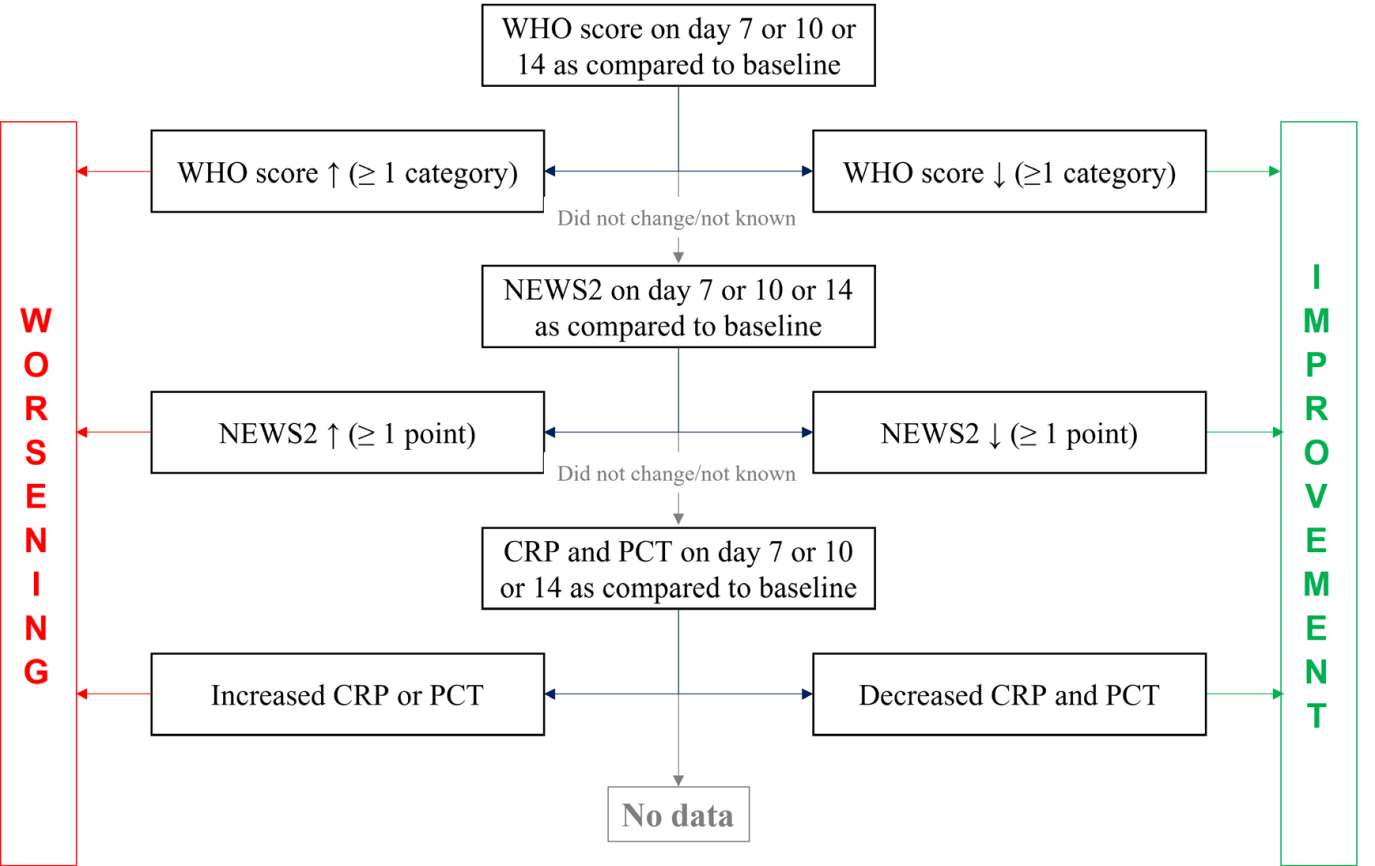
Definition of improvement and worsening at time points compared to baseline. WHO – World Health Organization, NEWS2 – National Early Warning Score 2, CRP – C-reactive protein, PCT – procalcitonin.

Secondary outcomes were as follows: a) orientation of discharge forming three distinct categories by severity: death, admittance to higher intensity care unit, and end of departmental stay (lower intensity care or home); and b) proportion of patients with clinical improvement on days 7, 10, and 14 compared to the baseline date. Algorithm for defining clinical improvement or worsening is shown in **Figure 2**. Laboratory changes were defined as at least 20% change in absolute values and both CRP and PCT had to decrease to be considered an improvement.

### Statistical analysis

Categorical variables are presented as absolute (n) and relative (%) frequencies, while quantitative variables are presented as mean ± standard deviation (SD). Two PSM cohorts were created by calculating propensity scores by logistic regression using the presence or absence of RDV therapy as the dependent variable, and four variables as covariates: sex, age, baseline NEWS2 score, and Charlson comorbidity score. The two cohorts were compared using the Pearson χ^2^ test for qualitative variables. Continuous variables were tested for normality (criteria determined by Hair et al. [17]) and compared using a student’s *t* test (in case of normal distributions) or a Mann-Whitney U test. Survival analysis was performed using Kaplan-Meier estimates. Participants were censored at day 61 or on the day of death. As an exploratory univariate analysis, relative risk (RR) was calculated for 60-day all-cause mortality and represented with 95% confidence intervals (CI) comparing the two treatment groups by subgroups. To measure the effect of RDV treatment on survival we calculated hazard ratios (HR) by performing a multivariate analysis using COX regression. The variables for COX regression were all parameters used in the exploratory univariate analysis. Age and Charlson score were included as ordinal variables. The interaction between age and Charlson score was included to compensate for the use of age in the calculation of Charlson score. Cox regression model was applied to those subgroups, which presented significant benefit in the univariate analysis, to calculate HR for RDV treatment. For each model, Harrell’s concordance index and Somers’ D statistic were calculated and displayed under the corresponding HRs, which are presented with 95% CIs.

The propensity score matching was performed using the SAS 9.4 (SAS Institute Inc., Cary, NC, USA) software package, while the SPSS software ver. 27.0.1.0. (IBM, Armonk, NY, USA) was used for all other analyses.

## RESULTS

In the observation period, 974 cases were admitted to COVID-19 wards at the Department of Pulmonology of Semmelweis University. Patient characteristics for all patients and Group RDV and Group SOC are summarized in **Table 2**. All patients were of Caucasian race. Significant differences have been observed between the two groups regarding baseline WHO scale, since RVD was only used in cases requiring supplementary oxygen. Furthermore, during the third wave of pandemic, all eligible patients received RDV resulting in significant difference in wave distribution. Malignancy and anaemia were more common in Group SOC, while diabetes and more extensive baseline parenchymal lung involvement were more common in Group RDV. Regarding laboratory parameters baseline CRP, Albumin, TP, Hgb, and RBC were significantly higher, while baseline ProBNP, GPT, PLT, and WBC were significantly lower in patients of Group RDV.

**Table 2:** Baseline patient characteristics

	All	Group RDV	Group SOC	*P*-value
	n = 974	n = 370	n = 370	
**Sex; n (%)**				
Female	436 (44.8%)	151 (40.8%)	151 (40.8%)	1.000
Male	538 (55.2%)	219 (59.2%)	219 (59.2%)	
**Wave; n (%)**				
2. wave	526 (54.0%)	134 (36.2%)	237 (64.1%)	<0.001
3. wave	448 (46.0%)	236 (63.8%)	133 (35.9%)	
**Age; years**	64.3 ± 15.17	62.2 ± 14.63	63.19 ± 15.92	0.375
**Age categories in years; n (%)**				
<55	251 (25.8%)	113 (30.5%)	103 (27.8%)	0.410
55-64	190 (19.5%)	82 (22.2%)	71 (19.2%)	
65-74	257 (26.4%)	90 (24.3%)	94 (25.4%)	
75≤	276 (28.3%)	85 (23.0%)	102 (27.6%)	
**WHO scale; n (%)**				
3 (Hospitalized)	209 (21.5%)	0 (0%)	57 (15.4%)	<0.001
4 (Supplementary O2)	740 (76%)	356 (96.2%)	306 (82.7%)	
5 (High-Flow O2)	24 (2.5%)	14 (3.8%)	7 (1.9%)	
**Charlson score; n (%)**				
0-3	486 (49.9%)	209 (56.5%)	204 (55.1%)	0.933
4-6	299 (30.7%)	111 (30.0%)	114 (30.8%)	
7≤	189 (19.4%)	50 (13.5%)	52 (14.1%)	
**Comorbidities; n (%)**				
Malignancy	134 (13.8%)	31 (8.4%)	44 (11.9%)	0.113
Hypertension	592 (60.8%)	220 (59.5%)	217 (58.6%)	0.823
Diabetes	275 (28.2%)	115 (31.1%)	81 (21.9%)	0.005
CAD	138 (14.2%)	46 (12.4%)	46 (12.4%)	1.000
Heart failure	157 (16.1%)	44 (11.9%)	55 (14.9%)	0.235
Asthma	73 (7.5%)	34 (9.2%)	22 (5.9%)	0.095
COPD	165 (16.9%)	54 (14.6%)	67 (18.1%)	0.196
Anaemia	102 (10.5%)	20 (5.4%)	44 (11.9%)	0.002
Dyslipidaemia	55 (5.6%)	18 (4.9%)	16 (4.3%)	0.726
**Parenchymal involvement on the initial CT, n (%)**				
<15%	383 (39.3%)	89 (24.1%)	189 (51.1%)	<0.001
15%-50%	354 (36.3%)	183 (49.5%)	107 (28.9%)	
50≤%	163 (16.7%)	84 (22.7%)	47 (12.7%)	
Missing	74 (7.6%)	14 (3.8%)	27 (7.3%)	
**Baseline parameters – mean**				
CRP	109.39 ± 85.93	126.08 ± 81.27	91.04 ± 88.07	<0.001
PCT	0.46 ± 1.81	0.41 ± 1.63	0.49 ± 2.09	0.601
Ferritin	1055 ± 1395	1141 ± 1114	1027 ± 1686	0.290
IL-6	83.48 ± 323.84	76.58 ± 129.05	77.65 ± 220.29	0.938
ProBNP	506.56 ± 1966.6	1181 ± 3509	1938 ± 4732	0.017
Troponin	32.19 ± 91.07	23.69 ± 61.23	29.62 ± 70.84	0.177
eGFR	71.19 ± 22.56	72.83 ± 19.69	72.53 ± 23.63	0.853
GOT	55.52 ± 77.94	56.63 ± 33.93	56.23 ± 114.98	0.950
GPT	46.31 ± 64.24	41.53 ± 26.68	52.89 ± 91.2	0.024
Albumin	31.86 ± 5.09	32.56 ± 4.21	31.43 ± 5.18	0.001
TP	62.56 ± 7.27	63.56 ± 6.45	61.97 ± 7.6	0.003
Hgb	128.98 ± 21.13	135.18 ± 18.01	126.32 ± 21.49	<0.001
RBC	4.34 ± 0.72	4.62 ± 0.63	4.33 ± 0.75	<0.001
PLT	257.12 ± 113.76	233.82 ± 95.06	268.83 ± 124.43	<0.001
WBC	8.70 ± 5.41	8.32 ± 5.59	9.42 ± 5.77	0.009
LC	1.26 ± 1.90	1.19 ± 1.9	1.26 ± 1.59	0.590

RDV – remdesivir, SOC – only standard of care, WHO – World Health Organization, CAD – coronary artery disease, COPD – chronic obstructive pulmonary disease, CRP – C-reactive protein, PCT – procalcitonin, IL-6 – interleukin-6, ProBNP – pro b-type natriuretic peptide, eGFR – estimated glomerular filtration rate, GOT – aspartate aminotransferase, GPT – alanin aminotransferase, TP – total protein, RBC – red blood cell count, Hgb – blood haemoglobin, PLT – platelet count, WBC – white blood cell count, LC – lymphocyte count

**Figure 3:**
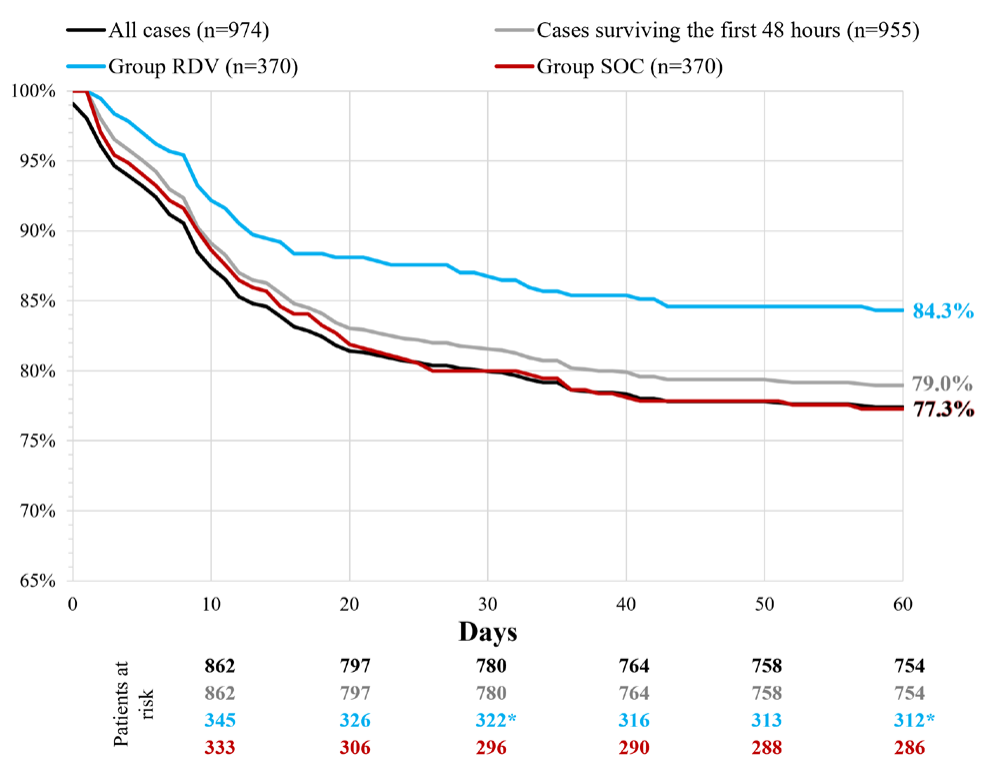
Kaplan-Meier curves of 60-day all-cause mortality. RDV – remdesivir, SOC – only standard of care. *Significantly better survival compared to Group SOC.

Outcome data are summarized in **Table 3**. RDV treatment significantly reduced 30- and 60-day all-cause mortality (**Figure 3**). Significantly less patients died in the RDV group, while higher dependency care was needed slightly more often. Short-term outcome for improvement at days 7, 10, and 14 compared to baseline did not differ in the two groups.

**Table 3:** Primary and secondary outcomes

	All cases	Group RDV	Group SOC	*P*-value
	n = 974	n = 370	n = 370	
**Primary outcomes**				
30-d all-cause mortality; n (%)	195 (20.0%)	49 (13.2%)	74 (20.0%)	0.014
60-d all-cause mortality; n (%)	220 (22.6%)	58 (15.7%)	84 (22.7%)	0.015
**Secondary outcomes**				
Orientation of discharge; n (%)				
Death	162 (16.6%)	36 (9.7%)	60 (16.2%)	0.031
Higher intensity care unit	91 (9.3%)	38 (10.3%)	33 (8.9%)	
Discharge	721 (74.0%)	296 (80.0%)	277 (74.9%)	
**Clinical improvement; n (%)**				
7 d after baseline	491 (50.4%)	191 (51.6%)	206 (55.7%)	0.269
10 d after baseline	586 (60.2%)	244 (65.9%)	239 (64.6%)	0.700
14 d after baseline	658 (67.6%)	273 (73.8%)	255 (68.9%)	0.143

RDV – remdesivir, SOC – only standard of care, d – days

**Figure 4:**
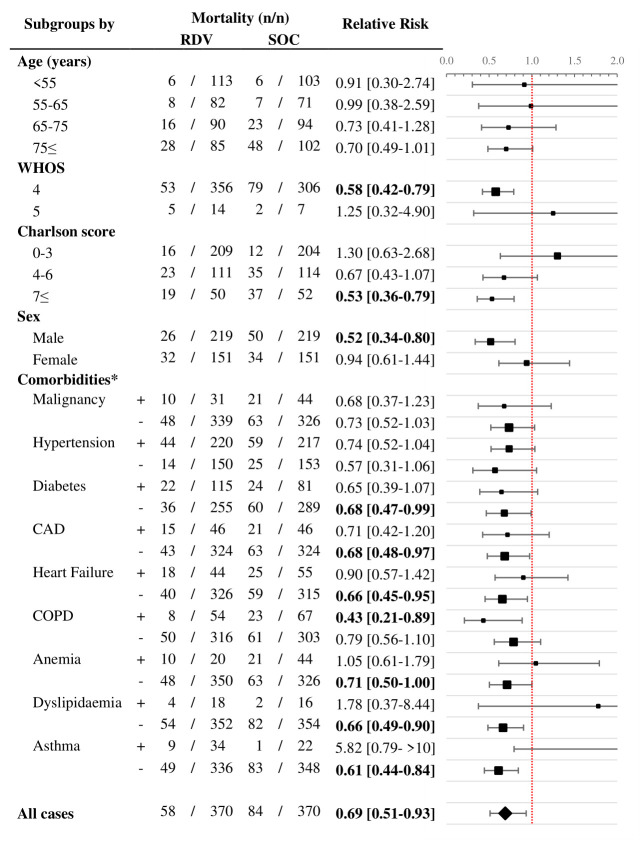
Relative risks comparing subgroups of Group RDV and Group SOC for 60-day all-cause mortality in a univariate analysis. RDV – remdesivir, SOC – only standard of care, WHOS – WHO’s ordinal scale for improvement, CAD – coronary artery disease, COPD – chronic obstructive pulmonary disease. *The symbol “+” signifies that a comorbid condition is present, while “−” signifies that a comorbid condition is absent.

**Figure 5:**
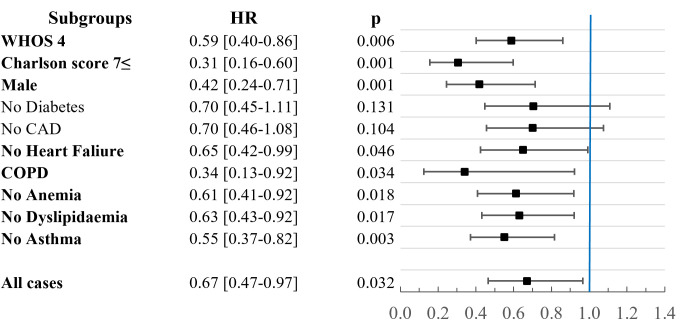
Hazard ratios for RDV use from the multivariate analyses in the subgroups. RDV – remdesivir, HR – hazard ratio, WHOS – WHO’s numerical scale for improvement, CAD – coronary artery disease, COPD – chronic obstructive pulmonary disease.

Treatment modalities used during the observation period (RDV and SOC only) are summarized in Figure S1 in the **Online Supplementary Document** showing the distribution of patients by special treatments (RDV, favipiravir, convalescent plasma therapy, other (baricitinib or tocilizumab)). Based on the univariate analysis for 60-day survival, male patients with WHOS 4, 7+ Charlson score, and COPD and patients without diabetes coronary artery disease, heart failure, anaemia, dyslipidaemia, and asthma benefited significantly from RDV use. There was no group where SOC alone was significantly beneficial. This is represented by the calculated relative risks by each subgroup in **Figure 4**. After performing the multivariate analyses for the groups which showed significant benefit, the advantage of RDV was further strengthened in the subgroups consisting of male patients with WHOS 4, Charlson score 7+, and COPD. Additionally, significant benefit for RDV use in groups without heart failure, anaemia, dyslipidaemia, and asthma was observed, possibly due to small number of patients presenting with these comorbidities. The calculated hazard ratios for all variables are presented in the Table S1 in the **Online Supplementary Document** and highlighting the HR for remdesivir by subgroups in **Figure 5**.

## DISCUSSION

Defining optimal treatment for COVID-19 patients, especially those with advanced age and multiple comorbidities, is challenging. Our data confirmed significant beneficial long-term outcome of 5-day RDV treatment in patients hospitalized for COVID-19 pneumonia in need of supplemental oxygen. Our most important finding was that antiviral RDV therapy significantly benefited men and patient with most comorbid conditions.

At the time of manuscript preparation, RDV is the only known antiviral drug of confirmed benefits in COVID-19. RDV received first conditional recommendation for 10-day treatment of hospitalized patients who require supplemental oxygen, but not mechanical ventilation [1]. Later studies also focused on 5-day RDV treatment, as no difference between a 5- and 10-day regimen was noted [18]. The 5-day course was an appropriate choice in our real-world setting, making discharge possible for patients who were fit for home and did not need a 10-day hospital stay only to complete the treatment. Additionally, therapy cost is also an important issue, making 5-day treatment an affordable choice for health care providers in middle- or low-income countries and making treatment accessible to more patients.

RDV was first approved for the treatment of COVID-19 patients needing supplementary oxygen due to pulmonary involvement; in our centre, all patients received RDV accordingly. According to the ACTT-1 trial, 10-day RDV treatment reduced the time to recovery in patients hospitalized with COVID-19 who had evidence of lower respiratory tract infection [1]. Using hospital stay as an outcome has significant limitations especially using long therapy course (eg, 10 days for RDV). However, several studies used length of hospital stay as an indicator of a successful treatment, but the difference in criteria used might have contributed to the overall difficulties of comprehensive conclusions [1,4]. Similar to our study, Ohl et al. analysed over 2000 propensity score-matched RDV treated and untreated US veterans. This study included mainly older participants, predominantly men, and confirmed longer hospital stay in RDV-treated patients, which might have been the result of variable (1 to 12 days) RDV treatment protocols [19]. This was the main reason that length of hospital stay was not included into our study; additionally, Hungarian laws regarding discharge criteria changed several times during the study period, making the parameter unusable as an indicator for improvement.

Rapid improvement of patients using a given treatment is always an important clinical outcome parameter. Meta-analysis of randomized controlled clinical trials of RDV treatment in 2020 showed major benefit for oxygen treated COVD-19 patients receiving RDV and defining favorable outcome as percentage of patients improving [20]. Our data did not confirm significant clinical improvement at 7, 10 and 14 days, similarly to the results of Ader et al in the DisCoVeRy phase 3 trial [21]. This difference might be mainly the result of the well-known difference between patient selection in randomized controlled trials and real-world data as well as the difference in the definition of clinical improvement. We created this new clinical improvement measure to compensate the fact that discharge from COVID wards was strictly linked to time spent in hospital by the Hungarian emergency regulations to decrease the spread of the virus by recovered but not virus free individuals.

In our real-world observational study men, patients over the age of 75 years and multimorbid patients benefited the most from RDV treatment. This is in sync with the results shown in the ACTT-1 clinical trial [1], which identified the most benefit in non-Hispanic Caucasians and patients older than 65 years compared to patients between 40 and 65 years [1]. As younger patients (<40 years) were underrepresented with 8% in out hospital cohort it is difficult to assess effectivity of treatment in this subgroup.

Our primary outcome did confirm significant benefit in view the reduction of 60-day mortality in RDV treated COVID-19 patients. Mortality is difficult to assess especially in multimorbid elderly patients. A large observational cohort study analyzing patients during the first wave did not confirm 30-day survival benefit for experimental 5-day RDV treatment [22]. Similarly to the ACTT-1 trial, a large retrospective observational multicentre trial focusing on data acquired from US health care database with patients hospitalized between August and November 2020 showed the largest benefit of RDV therapy in patients receiving low-flow supplemental oxygen. The 28-day survival rate of RDV and non-RDV patients in the low-flow supplemental oxygen group (89.3% and 84.9%, respectively) of this study is comparable to our results (87.0% and 80.0%, respectively), while our observation was extended to 60 -day and longer-term survival benefit was additionally confirmed.

Multimorbidity is a significant risk factor for death and the Charlson comorbidity score is widely used as a prognostic factor for prediction of all-cause mortality [23]. A study by Russo et al. [24] reporting real-world COVID-19 outcome data from an Italian hospital observed marginal, but not significant 30-day survival benefit comparing two PSM cohorts comprising patients with similar average age (over 60 years) but with a lower average Charlson score compared to our cohorts (RDV = 2.6, non-RDV = 2.5 for Russo et al.; RDV = 3.4, non-RDV = 3.5 for our study). A real-world observational study from Granada focusing on RDV treated patients also showed an increased survival rate (89.7%) compared to the global mortality rate of COVID-19 cases in the clinical centre (79.7%). This smaller study population had similar baseline characteristics as our RDV cohort, including age and Charlson comorbidity score [25]. However, patients over the age of 70 years have a Charlson score of at least 3, so our data showing the greatest benefit of RDV treatment in patients with Charlson score >7 included mainly elderly with several comorbid conditions or younger patients with very severe diseases. This is a new and important finding, as no adverse events associated with RDV treatment were registered, making this antiviral treatment safe for the most vulnerable patient population.

An additional novelty of our analysis is that in COPD, an important and prevalent comorbid condition, RDV treatment was significantly advantageous as compared to SOC therapy. Similarly, men did significantly profit over long-term from antiviral treatment. These variables were independently associated with decreased mortality following RDV treatment, defining a well-defined subgroup of patients.

Age is an important predictor of mortality [26]. However, our data did not confirm a significant therapeutic benefit of RDV in elderly. However, RDV associated better outcome did increase with age. Another novelty of our data are the inclusion of lung parenchymal involvement into the analysis. Our previous study did confirm that artificial intelligence-based lung CT severity assessment by parenchymal involvement showed effect on outcome [27], and this new analysis added the therapeutic advantage of RDV-treated patients with higher parenchymal involvement.

The strengths of this study are the long-term outcome analysis in a real-world setting, including several aspects of the rapidly changing pandemic, such as differences in discharge criteria, treatment protocols, virus subtypes, and vaccination of health care professionals, and high number of standardized patient care, making propensity score matching possible.

The study limitations are the protocol change during third wave as all eligible patients got RDV, which could have distorted the results even after propensity score matching; the change of discharge criteria in November 2020, as patients with clinical and radiological improvement could go to home isolation without the previously needed two negative consecutive PCR results within 48 hours, making length of stay calculation inconsistent during our study; the inclusion of patients from only one hospital, as different protocols can influence the outcome; the use of all-cause mortality as an outcome, as remdesivir supposedly only decrease COVID-19 associated mortality. Lastly a strong limiting factor for our subgroups analysis is the low number of patients presenting with specific comorbid conditions.

The analysis of suggested beneficial groups should be examined in later studies. A possible next step could be a retrospective meta-analysis focusing on the use of RDV in COPD patients as well as a study investigating if the proposed benefit of RDV use in men could be observed in other real-world populations.

## CONCLUSIONS

Our data confirmed a 30- and 60-day mortality benefit following 5-day RDV treatment in severe COVID-19 pneumonia. According to our data, more severe cases, especially men, COPD patients and patients with multiple comorbidities in the need of oxygen support, should receive 5-day RDV as better long-term outcome can be assumed. The study did not confirm short-term beneficial effects of RDV, as improvement at days 7, 10, and 14 were not different according to treatment protocols.

## Additional material


Online Supplementary Document
