# Supplementary material for: Long-term survival benefit of male and multimorbid COVID-19 patients with 5-day remdesivir treatment
Source: J Glob Health. 2022 Aug 31;12:05031. doi: 10.7189/jogh.12.05031 (PMC9428504; doi:10.7189/jogh.12.05031)
Supplement: Online Supplementary Document [file jogh-12-05031-s001.pdf]

Supplementary Figure 1. Use of treatment modalities in the observed population during the studied period.

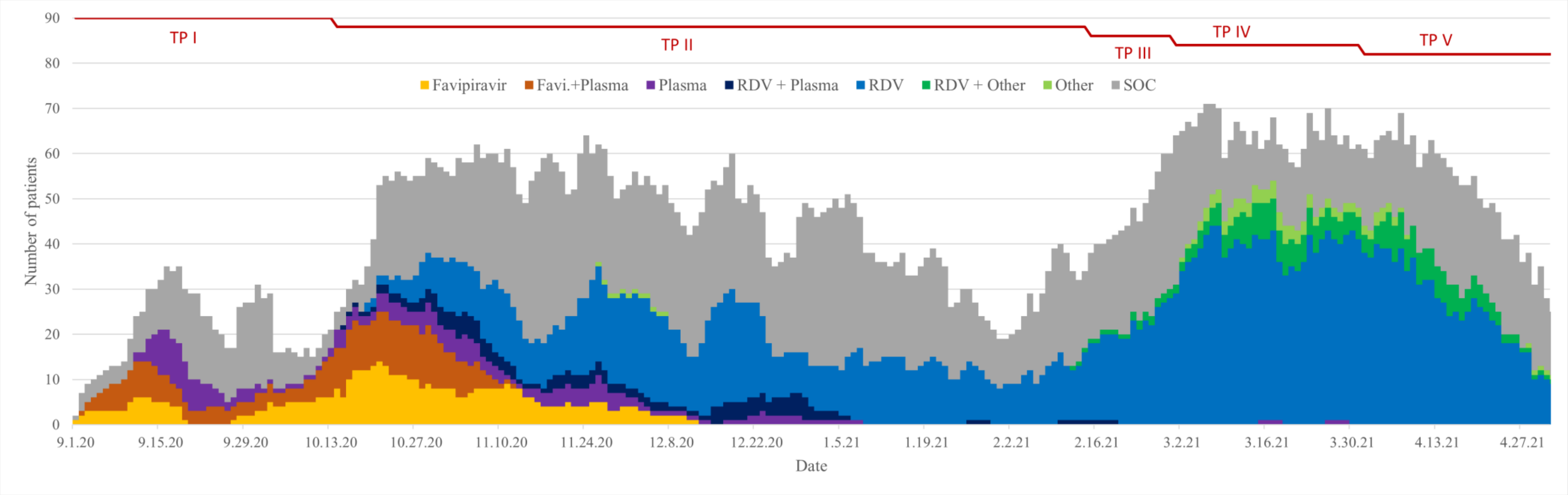

TP: treatment protocols according Table 1, RDV: remdesivir, SOC: only standard of care

Supplementary Table 1. Calculated Hazard Ratios in

	WHOS 4	Charlson score 7+	Male	No Diabetes	No CAD	No Heart Failure	COPD	No Anemia	No Dyslipid.	No Asthma	All cases
Male sex	1.10 [0.77-1.57]	1.07 [0.60-1.90]		0.99 [0.66-1.50]	0.90 [0.61-1.35]	1.13 [0.75-1.70]	0.60 [0.26-1.40]	1.23 [0.83-1.81]	1.09 [0.77-1.54]	1.04 [0.73-1.48]	1.03 [0.73-1.45]
Age (+1 year)	1.04 [1.01-1.08]*	1.13 [0.96-1.33]	1.04 [0.99-1.08]	1.06 [1.01-1.10]*	1.03 [0.99-1.07]	1.05 [1.01-1.10]*	1.04 [0.91-1.19]	1.05 [1.01-1.10]*	1.05 [1.01-1.08]*	1.04 [1.01-1.08]*	1.05 [1.01-1.08]*
Charlson score (+1 point)	2.43 [1.58-3.74]*	4.19 [0.82- >10]	2.34 [1.35-4.04]*	2.82 [1.65-4.83]*	1.94 [1.22-3.09]*	2.81 [1.69-4.67]*	5.42 [1.25- >10]*	2.30 [1.38-3.84]*	2.25 [1.47-3.44]*	2.17 [1.42-3.32]*	2.28 [1.50-3.46]*
Age*Charlson score (interaction)	0.99 [0.99-1.00]*	0.99 [0.97-1.01]	0.99 [0.99-1.00]	0.99 [0.98-1.00]*	1.00 [0.99-1.00]	0.99 [0.98-1.00]*	0.99 [0.97-1.01]	0.99 [0.99-1.00]	0.99 [0.99-1.00]*	0.99 [0.99-1.00]	0.99 [0.99-1.00]*
Wave 2	1.20 [0.81-1.77]	1.08 [0.55-2.11]	1.17 [0.68-2.01]	1.00 [0.63-1.58]	1.18 [0.76-1.84]	1.12 [0.72-1.74]	1.10 [0.45-2.66]	1.09 [0.71-1.68]	1.08 [0.74-1.60]	1.07 [0.72-1.59]	1.05 [0.72-1.52]
Malignancy	0.65 [0.33-1.27]	0.57 [0.22-1.52]	0.69 [0.30-1.60]	0.74 [0.33-1.69]	0.96 [0.47-1.93]	0.57 [0.27-1.21]	0.14 [0.03-0.74]*	0.85 [0.39-1.86]	0.79 [0.41-1.52]	0.76 [0.40-1.45]	0.72 [0.38-1.36]
Hypertension	1.05 [0.69-1.60]	1.06 [0.47-2.39]	0.75 [0.45-1.26]	1.19 [0.75-1.88]	1.11 [0.71-1.76]	1.01 [0.63-1.62]	0.85 [0.28-2.57]	1.09 [0.69-1.71]	1.06 [0.70-1.61]	1.12 [0.73-1.70]	1.01 [0.67-1.52]
Diabetes	0.50 [0.31-0.82]*	0.70 [0.35-1.43]	0.64 [0.33-1.27]		0.52 [0.30-0.90]*	0.62 [0.34-1.13]	0.27 [0.08-0.93]*	0.46 [0.27-0.80]*	0.56 [0.34-0.91]*	0.58 [0.35-0.96]*	0.55 [0.34-0.89]*
Coronary artery disease	0.71 [0.45-1.14]	0.44 [0.23-0.84]*	1.30 [0.71-2.41]	0.91 [0.53-1.57]		0.84 [0.47-1.5]	0.49 [0.17-1.44]	0.62 [0.35-1.08]	0.81 [0.51-1.29]	0.74 [0.46-1.19]	0.76 [0.48-1.20]
Heart Failure	1.10 [0.70-1.72]	1.42 [0.77-2.62]	0.90 [0.50-1.64]	1.25 [0.72-2.16]	1.06 [0.64-1.77]		0.50 [0.18-1.40]	1.25 [0.75-2.07]	1.17 [0.76-1.80]	1.06 [0.68-1.64]	1.16 [0.76-1.77]
COPD	0.78 [0.50-1.21]	0.58 [0.29-1.14]	0.37 [0.18-0.76]*	0.71 [0.42-1.20]	0.64 [0.37-1.12]	0.75 [0.43-1.31]		0.59 [0.35-1.01]	0.68 [0.43-1.07]	0.67 [0.42-1.06]	0.71 [0.46-1.10]
Anemia	1.01 [0.63-1.62]	0.97 [0.49-1.91]	0.76 [0.39-1.49]	0.81 [0.44-1.47]	0.74 [0.41-1.33]	1.42 [0.81-2.52]	1.78 [0.59-5.36]		1.12 [0.71-1.78]	1.07 [0.67-1.71]	1.16 [0.74-1.80]
Dyslipidemia	0.41 [0.16-1.04]	0.29 [0.08-1.00]*	0.24 [0.06-1.04]	0.49 [0.12-2.07]	0.71 [0.28-1.83]	0.37 [0.13-1.08]	0.96 [0.28-3.27]	0.54 [0.19-1.5]		0.34 [0.12-0.95]*	0.49 [0.21-1.14]
Asthma	1.80 [0.85-3.81]	0.00 [0.00- >10]	3.1 [0.88-10.89]	2.34 [1.01-5.44]*	1.83 [0.85-3.94]	1.14 [0.41-3.20]	2.32 [0.72-7.53]	1.92 [0.85-4.31]	1.63 [0.77-3.45]		1.88 [0.95-3.70]
Remdesivir	0.59 [0.40-0.86]*	0.31 [0.16-0.60]*	0.42 [0.24-0.71]*	0.70 [0.45-1.11]	0.70 [0.46-1.08]	0.65 [0.42-0.99]*	0.34 [0.13-0.92]*	0.61 [0.41-0.92]*	0.63 [0.43-0.92]*	0.55 [0.37-0.82]*	0.67 [0.47-0.97]*
Harrell's C	0.8045	0.7393	0.8055	0.8198	0.8154	0.7897	0.8273	0.8035	0.8038	0.7992	0.7998
Somers' d	0.6090	0.4787	0.6110	0.6397	0.6309	0.5795	0.6545	0.6071	0.6076	0.5984	0.5995

WHOS: WHO’s numerical scale for improvement, CAD: coronary artery disease, COPD: chronic obstructive pulmonary disease

*significant ($p < 0.05$) Hazard Ratio
